# Laser evoked potentials and central sensitization in migraine

**DOI:** 10.1186/1129-2377-16-S1-A17

**Published:** 2015-09-28

**Authors:** Eleonora Vecchio, Vittorio Sciruicchio, Katia Ricci, Anna Montemurno, Marianna Delussi, Marina de Tommaso

**Affiliations:** Neurophysiopathology of Pain Unit, University “Aldo Moro”, Bari, Italy; Pediatric Neurology Division, Bari Policlinico General Hospital, Bari, Italy

Migraine is a disabling disorder of neuro-vascular origin. An abnormal neuronal excitability, largely based on genetic nature, is a predisposing factor to attack onset. A reduced habituation to multimodal repetitive non nociceptive stimuli was observed in migraine. The pattern of reduced habituation to nociceptive stimuli may favor the increase of pain and the phenomena of central sensitization. Central sensitization is a phenomenon of pain processing, which may predispose to chronic pain. Allodynia occurring during migraine attack and persistent pericranial tenderness in migraine are symptoms of central sensitization[1, 2]. CO2 laser evoked potentials (LEPs) have been used in migraine research, proving very useful in demonstrating functional abnormalities of the central nociceptive system which might be linked to the pathophysiological mechanisms of this disease. Abnormalities of pain processing seem to characterize children with migraine. Reduced habituation and progressive amplification of cortical responses under laser stimuli indicate an overactive nociceptive system just at the onset of migraine, which may subtend symptoms of central sensitization as allodynia and pericranial tenderness. An abnormal pattern of habituation of the trigeminal nociceptive system is an endophenotypic marker of migraine[3], suggesting that the nociceptive system is prone to sensitization even before clinical appearance[4]. These aspects may guide therapeutic approach: in a recent work in which we explored the efficacy of botulinum toxin in the treatment of chronic migraine, we found a correlation of the clinical effect with an improvement of the LEPs habituation deficit. The modes of action of pharmacological or nonpharmacological interventions, such as neuromodulation methods, should therefore be reconsidered in terms of their ability to normalize the complex abnormalities of brain hyperresponsivity and the central sensitization phenomena.
